# Comparative distributions of RSBN1 and methylated histone H4 Lysine 20 in the mouse spermatogenesis

**DOI:** 10.1371/journal.pone.0253897

**Published:** 2021-06-29

**Authors:** Youtao Wang, Tokuko Iwamori, Takane Kaneko, Hiroshi Iida, Naoki Iwamori

**Affiliations:** 1 Laboratory of Zoology, Graduate School of Bioresource and Bioenvironmental Sciences, Kyushu University, Fukuoka-shi, Fukuoka, Japan; 2 Laboratory of Zoology, Graduate School of Agriculture, Kyushu University, Fukuoka-shi, Fukuoka, Japan; Centre de Recherche en Cancerologie de Lyon, FRANCE

## Abstract

During spermatogenesis, nuclear architecture of male germ cells is dynamically changed and epigenetic modifications, in particular methylation of histones, highly contribute to its regulation as well as differentiation of male germ cells. Although several methyltransferases and demethylases for histone H3 are involved in the regulation of spermatogenesis, roles of either histone H4 lysine 20 (H4K20) methyltransferases or H4K20 demethylases during spermatogenesis still remain to be elucidated. Recently, RSBN1 which is a testis-specific gene expressed in round spermatids was identified as a demethylase for dimethyl H4K20. In this study, therefore, we confirm the demethylase function of RSBN1 and compare distributions between RSBN1 and methylated H4K20 in the seminiferous tubules. Unlike previous report, expression analyses for RSBN1 reveal that RSBN1 is not a testis-specific gene and is expressed not only in round spermatids but also in elongated spermatids. In addition, RSBN1 can demethylate not only dimethyl H4K20 but also trimethyl H4K20 and could convert both dimethyl H4K20 and trimethyl H4K20 into monomethyl H4K20. When distribution pattern of RSBN1 in the seminiferous tubule is compared to that of methylated H4K20, both dimethyl H4K20 and trimethyl H4K20 but not monomethyl H4K20 are disappeared from RSBN1 positive germ cells, suggesting that testis-specific distribution patterns of methylated H4K20 might be constructed by RSBN1. Thus, novel expression and function of RSBN1 could be useful to comprehend epigenetic regulation during spermatogenesis.

## Introduction

Spermatogenesis is the process to form spermatozoa in the seminiferous tubules of the testis. Starting with the proliferation stage, mitotic divisions cause spermatogonia, which located on the basement membrane of the tubules, to differentiate into spermatocytes. In the meiotic stage, each spermatocyte is further divided into four haploid spermatids, and chromatin of germ cells undergoes a series of drastic changes including chromosome condensation, chromosome synapsis, homologous recombination and chromosome separation. The round spermatids produced from spermatocyte are then transformed into elongated spermatids to form spermatozoa through acrosome formation, chromatin compaction, tail formation, as well as phagocytosis at the stage of spermiogenesis [1,2].

Nucleosome is the core structure of chromatin. It contains a histone octamer and a double-strand DNA which wraps around histone octamer, each octamer consists of two copies of four histone proteins: H2A, H2B, H3, and H4 [3–5]. N-terminus of each histone proteins can be modified by some chemical groups, such as acetyl group (acetylation), methyl group (methylation), and so on. Each modification plays a vital role in modulating chromatin structure and affecting transcriptional regulation activity. For example, several lysines (K) of H3 and H4, such as H3K4, H3K9, H3K27, H3K36, and H4K20, could be modulated through three different methylation status: mono-methylation (me1), di-methylation (me2) and tri-methylation (me3). The methylated H3K4, H3K36, and H3K79 facilitate transcription, whereas methylated H3K9, H3K27, and H4K20 repress transcriptional activity [6–9].

Among several methylated histones, methylated H4K20 is unique to oscillate during the cell cycle and has multiple functions other than transcriptional repression [10–12]. Methylated H4K20 is critical for genome integrity including DNA damage repair, DNA replication, and chromatin compaction [13–18]. The function of methylated H4K20 is varied among methylated degree. H4K20me1 has function during both active transcription and transcriptional repression [19–21]. Moreover, it is also involved in X-chromosome dosage compensation in *C*. *elegans* [22–25]. H4K20me3 is essential for the silencing of repetitive DNA and retrotransposons as well as the regulation of telomere length [15,19,26,27]. Although distributions of methylated H4K20 during spermatogenesis was shown, the roles of methylated H4K20 during spermatogenesis still remain to be elucidated [28,29].

In mammals, at least three methyltransferases (PR-SET7/SET8/KMT5A, SUV420H1/KMT5B, and SUV420H2/KMT5C) and at least three demethylases (PHF8/KDM7B, LSD1n, and RSBN1/KDM9) directly regulate H4K20 methylation [10,12,15,22,30–32]. PR-SET7 catalyzes mono-methylation of H4K20, and SUV420 H1 and SUB420H2 catalyze di- and tri-methylation of H4K20. On the other hand, PHF8 can remove monomethyl mark from H4K20, whereas LSD1n and RSBN1 can demethylate dimethyl H4K20. Although some modifiers of histone H3 methylation are shown to play a role during spermatogenesis, roles of either H4K20 methyltransferases or H4K20 demethylases during spermatogenesis still remain unclear [33–38].

Among several modifiers for the methyl group of H4K20, RSBN1 (Round spermatid basic protein 1) was found as a testis-specific gene, which was specifically expressed in round spermatids [39]. Moreover, it was shown that RSBN1 could convert H4K20me2 but not H4K20me3 to H4K20me1 [22]. In this study, we re-examined the demethylation function of RSBN1 as well as the localization of RSBN1 in the seminiferous tubule and compared the distribution pattern between RSBN1 and methylated H4K20 during spermatogenesis.

## Materials and methods

### Semiquantitative RT-PCR

Total RNA of twelve different tissues isolated from three-months-old ICR mice were extracted by ISOGEN II (Nippon Gene), followed by reverse transcription using PrimeScript II RTase and random primer (Takara). The primers to amplify *Rsbn1* cDNA fragment spanning exons 2 and 7 were as follows: forward, 5’-AGTGAAAATGAGAAAAACGC-3’; reverse, 5’-CACAGGAGTGCTT GGATGTG-3’. PCR amplifications were repeated with three independent samples.

### Cell culture and transfection

The open reading frame (ORF) sequences of green fluorescence protein (GFP) and mouse RSBN1 was subcloned into pCS2-F vector, which contains a C-terminal FLAG tag sequence, and pCAGGS vector by In-fusion HD cloning kit (Takara) according to the manufacturer’s instruction. HeLa cells were maintained in Dulbecco’s modified Eagle Medium (Thermo scientific) supplemented with 10% fetal calf serum (SIGMA), 1% glutamine (Wako), and penicillin-streptomycin (Wako) at 37 ˚C in a humidified 5% CO2 atmosphere in air. The cells were transfected using Screen*F*ect^TM^ A *plus* (Wako) following manufacturer’s instruction. HeLa cells were cultured for 24 hours and re-seeded onto poly-L-lysine coated coverslips, followed by immunostaining analysis. For histone extraction, HeLa cells were electroporated by the pCS2F-GFP-RSBN1 using NEPA21 (Nepa Gene) according to manufacturer’s instruction. All transfections were repeated at least three times.

### Histone extraction and western blot

To detect histone modifications, histone extraction followed by immunoblot was performed as previously described [40]. Briefly, the nuclear fraction was first collected after cell lysis in the following buffer: 100mM Tris/HCl [pH 7.5], 1.5M NaCl, 1.5mM MgCl_2_, 0.65% NP-40, and protease inhibitor cocktail. Histones were extracted from the nuclear fraction by treatment with 0.2M H_2_SO_4_, followed by trichloro-acetic acid agglutination and immunoblot analysis. Histones isolated from ten thousand cells were separated by SDS-PAGE. To detect RSBN1 protein in multiple tissues isolated from three-months-old ICR mice or culture cells, RIPA lysis buffer (10mM Tris-HCl [PH7.4], 150mM NaCl, 0.5M EDTA, 1% NP-40,0.1% SDS) was utilized to prepare tissue lysate samples and cell lysates. Ten μg of tissue lysate were separated by SDS-PAGE. An antibody against mouse RSBN1 was developed in a rabbit by an immunization with the RSBN1 specific peptide (SCRUM). The sequence of RSBN1 specific peptide is as follows: Cys-ETAQNTESNSNM. Other primary antibodies used are as follows: anti-GFP (MBL), anti-Flag (Wako), anti-H4K20me1 (GeneTex and Wako), anti-H4K20me2 (GeneTex and Wako), anti-H4K20me3 (Abcam and Wako) as well as anti-histone H4 (Millpore) antibodies. Secondary antibodies are as follows: Goat anti-rabbit IgG-HRP and Goat anti-mouse IgG-HRP (both from Bio-Rad). To compare amounts of methylated H4K20, band densities of methylated H4K20 in three independent experiments were quantified by Quantity One software (Bio-Rad), and relative fold change of histones of RSBN1-transfected cells compared to control cells was presented as the mean ± SEM. All mouse experiments were performed in a accordance with protocol approved by the Institutional Animal Care and the Ethics Committee on Animal Experiments at Kyushu University (A30-025-0, A20-159-0).

### Immunofluorescence

At the 72 hours after transfection, the cells were fixed by 4% paraformaldehyde for 15 min, permeabilized with 0.5% triton X-100 for 20 min, washed, and immunostained by the methylated H4K20 antibodies described above and the Alexa 555-conjugated anti-rabbit IgG (Thermo Fisher Scientific) before examination by fluorescence microscopy.

Two- to three-months-old mouse testes were fixed in 4% paraformaldehyde (PFA) for 48 hours, followed by infiltration in 30% sucrose solution for overnight, and then testes were embedded by OCT compound (Sakura Finetek Japan) and frozen by liquid nitrogen. Frozen sections (6μm sickness) were cut and placed on adhesive glass slides. After rinsed in PBS and permeabilized with 0.5% triton X-100, samples of testis and cell were blocked in 3% bovine serum albumin (BSA)-PBS for one hour at room temperature. Then, samples were incubated with primary antibodies, which were diluted 1:1,000 with 3% BSA-PBS blocking buffer, for overnight at 4˚C, followed by incubation with secondary antibodies for one hour at room temperature. Primary antibodies used in this study were described above. Secondary antibodies were Alexa 555-conjugated donkey anti-rabbit IgG and Alexa 488-conjugated goat anti-mouse IgG (both from Thermo Fisher Scientific). The samples were examined by fluorescence microscopy after nuclear staining with DAPI and acrosome staining with PNA. Stages of seminiferous epithelium were determined by acrosome staining.

## Results

### Distribution of H4K20me3/me2/me1 demonstrates different pattern

**Fig 1:**
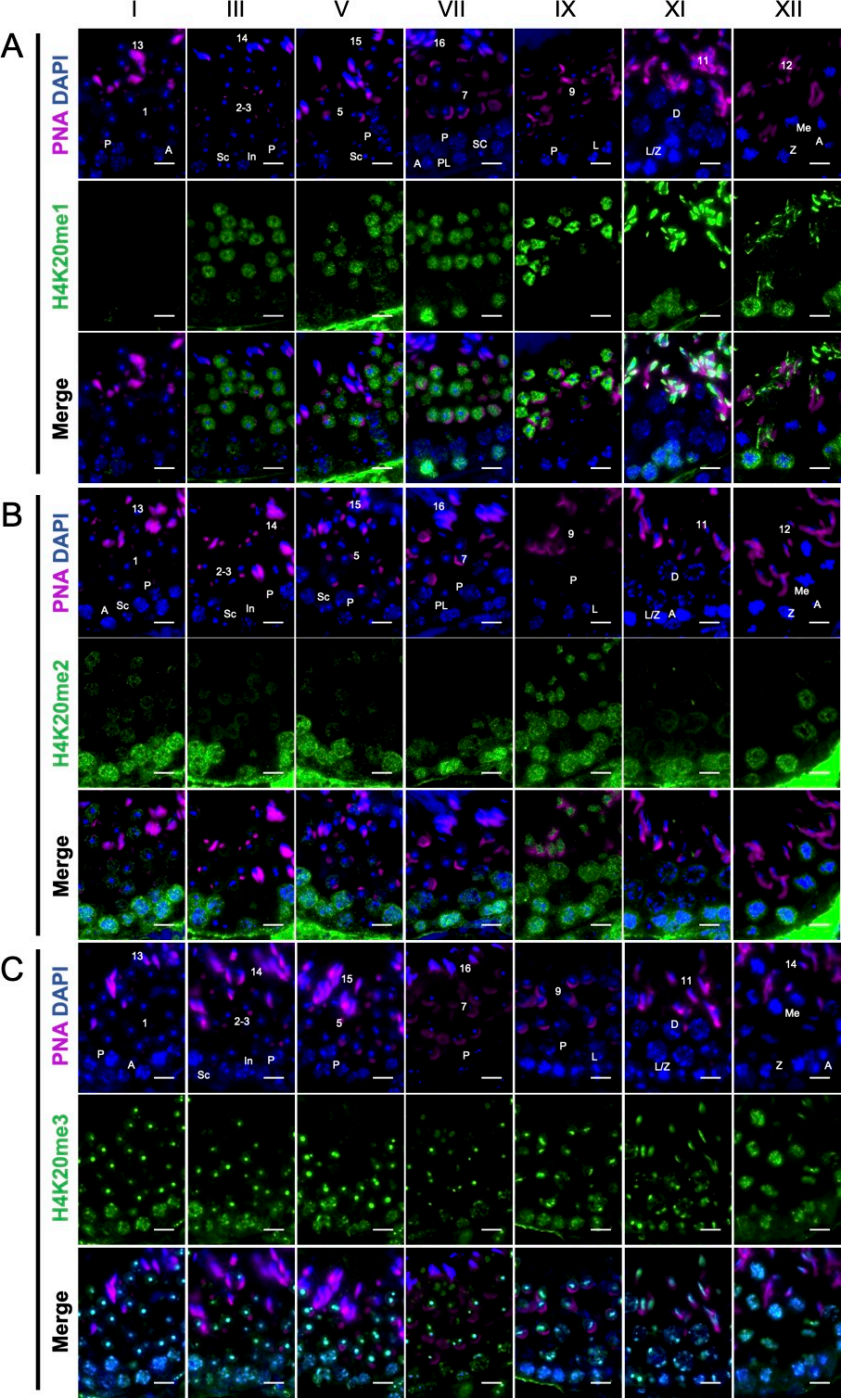
The distribution of trimethylated H4K20 in the seminiferous tubule. The distributions of nucleus and acrosomes (DAPI, blue; PNA, magenta: Top panels), tri-methylated (A), di-methylated (B), and mono-methylated (C) H4K20 (green: Middle panels), and merged images (bottom panels) in the stages I, III, V, VII, IX, XI, and XII are shown. A, type A spermatogonia; PL, preleptotene spermatocyte; L, leptotene spermatocyte; Z, zygotene spermatocyte; P, pachytene spermatocyte; Sc, Sertoli cells; Numbers, step of post-meiotic germ cells. Scale bars = 10μm.

In order to explore distribution patterns of H4K20me3, H4K20me2, and H4K20me1 during spermatogenesis, respectively, immunofluorescence experiments were performed. The strong H4K20me1 signal was detected in post-meiotic germ cells at the stages III through XII but not in round spermatids at the stage I, whereas the H4K20me1 signal was detected in spermatogonia and zygotene spermatocytes but not in pachytene spermatocytes (Fig 1A). H4K20me2 was strong in pre-leptotene and zygotene spermatocytes, moderate in pachytene spermatocytes, and weak in round spermatids at the stages I to V (Fig 1B). Although H4K20me2 signal was not detected in round and elongating spermatids and remained strong in pre-leptotene and moderate in pachytene spermatocytes at the stage VII, H4K20me2 was temporarily detected in elongating spermatids at the stage IX and disappeared from elongating spermatids again at the stages XI and XII (Fig 1B). At the stage XII, H4K20me2 was strong in zygotene spermatocytes and was moderately accumulated at meiotic cells (Fig 1B). On the other hand, H4K20me3 was formed strong foci at the heterochromatic region of round spermatids and was moderate in pachytene spermatocyte (Fig 1C). H4K20me3 foci were kept in round spermatids from the stage I to the stage V but became weak at the stage VII (Fig 1C). At the stages IX to XII, the strong foci of H4K20me3 were kept in chromosomes of elongating spermatids and positive signals of H4K20me3 were detected in pachytene and zygotene spermatocytes and meiotic chromosomes (Fig 1C).

### RSBN1 expression in multiple tissues and its localization in the testis

**Fig 2:**
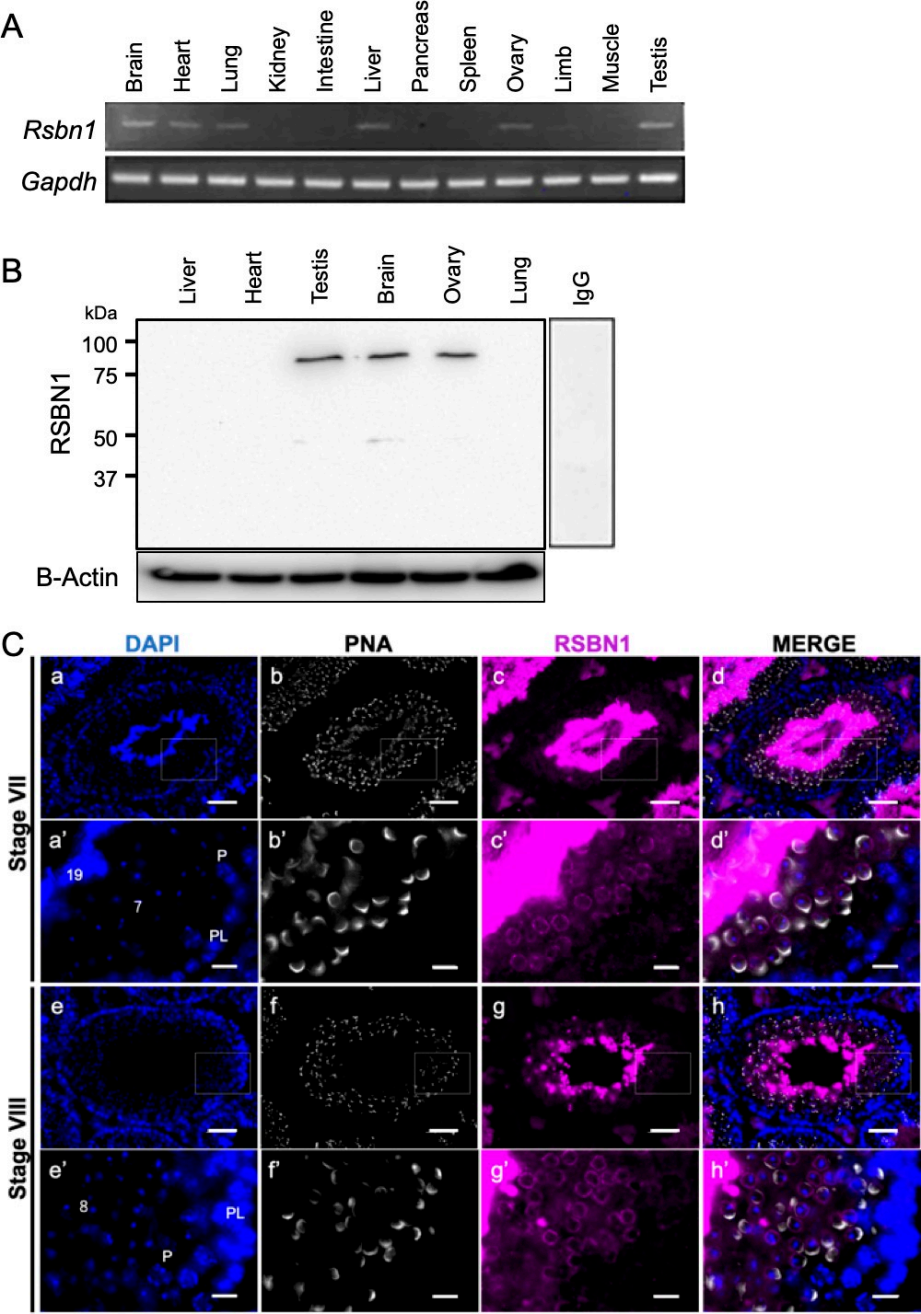
Expression and localization of RSBN1 in the testis. (A) Expression of *Rsbn1* mRNA in multiple tissues. Transcripts of *Rsbn1* were examined by semiquantitative RT-PCR in different tissue samples, *GAPDH* was used as an internal control. (B) Expression of RSBN1 protein in multiple tissues. A western blot results of RSBN1 protein in six different tissues are shown. Beta-actin was used as a loading control. (C) Localization of RSBN1 in the seminiferous tubules. Low magnification images (a-h) and high magnification images (a’-h’) of the localization of nucleus (DAPI, blue: a, a’, e, and e’), acrosome (PNA, white: b, b’, f, and f’), RSBN1 (magenta: c, c’, g, and g’), and merged images (d, d’, h, and h’) in the seminiferous tubule are shown. Scale bars = 50μm (a-h) and 10μm (a’-h’).

RSBN1 was previously found to be expressed in round spermatids as a testis specific gene. To confirm the expression of RSBN1, its expression in multiple adult mouse tissues was examined. RSBN1 transcripts were detected not only in testis but also in other tissues. RT-PCR suggested that RSBN1 was expressed at high level in testis and brain, at moderate level in heart, lung, liver, and ovary, and at low level in limb (Fig 2A). The RT-PCR results showed similar expression levels in public databases (ENCODE and EMBL-EBI). To detect the expression of RSBN1 products, an antibody against mouse RSBN1 was developed and its specificity was determined by immunoblotting and immunostaining, in which RSBN1 fused with GFP on N-terminus and with FLAG tag on C-terminus was used for the evaluation (S1 and S2 Figs). The lysates extracted from six different tissues, which include brain, testis, ovary, liver, lung, and heart, were analyzed by an immunoblot. The results demonstrated that RSBN1 protein was expressed in testis, brain, and ovary, but not in liver, heart, and lung (Fig 2B). Although an immunofluorescence of testis sections indicated that RSBN1 protein signals were mainly found in whole of elongated spermatids, RSBN1 was detected in the perinuclear region of round spermatids at the stage VII and VIII (Fig 2C) Whereas an intensity of RSBN1 in round spermatids was weak, that in elongated spermatids was quite strong (Figs 2C and S3).

### RSBN1 exhibits demethylation function on H4K20me3 and H4K20me2

**Fig 3:**
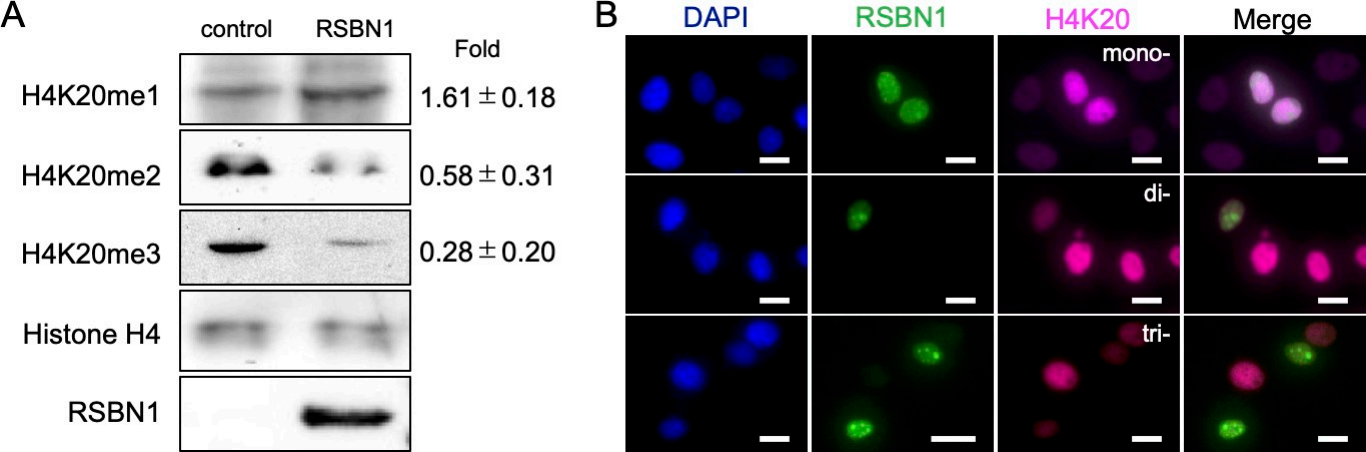
Demethylation activity of RSBN1. (A) Immunoblot results of methylated H4K20 in non-transfected cells (control) and transfected cells (*Rsbn1*). Values of relative Fold Change of band density (RSBN1/Control) are shown at right of each images. (B) An effect of RSBN1 expression on methylated H4K20. Immunostaining images of nucleus (DAPI, blue), RSBN1 (green), tri-methylated (magenta: Top), di-methylated (magenta: Middle), and mono-methylated (magenta: Bottom) H4K20 examined in RSBN1 expressing HeLa cells are shown. Scale bars = 20μm.

To evaluate the function of mouse RSBN1 and whether it can demethylate mono-, di-, trimethylated H4K20, RSBN1 was expressed in HeLa cells, and the methylation status of histone H4 was examined. RSBN1 was mainly localized in nucleus but not in cytoplasm of cells. H4K20me3 and H4K20me2 were significantly decreased with RSBN1 expression, whereas H4K20me1 was increased in RSBN1-expressing cells by immunoblot analysis (Fig 3A). Density of H4K20me3 and H4K20me2 bands in RSBN1-expressing cells was decreased at 0.28±0.2-fold and at 0.58±0.31-fold, respectively, whereas H4K20me1 density was increased at 1.61±0.18-fold in RSBN1-expressing cells (Fig 3A). Results were consistent with those obtained by immunostaining experiments (Fig 3B). These results suggested that RSBN1 can demethylate not only H4K20me2 but also H4K20me3 into H4K20me1.

**Fig 4:**
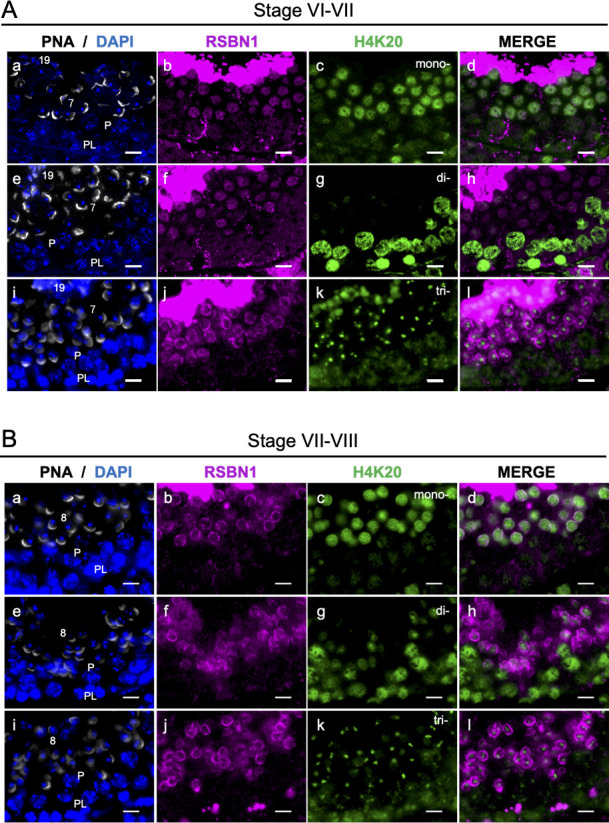
Distributions of methylated H4K20 and RSBN1 in the stage VIII of the seminiferous tubule. The distribution of nucleus and acrosome (DAPI, blue; PNA, white: a, e, i) and immunostained images of RSBN1 (magenta: b, f, j) and methylated H4K20 (green: c, g, k) in the stage VI-VII (A) and the stage VII-VIII (B) seminiferous tubules are shown. PL, pre-leptotene spermatocyte; P, pachytene spermatocyte; Numbers, steps of spermatids. Scale bars = 10μm.

Next, distributions of RSBN1 and methylated H4K20 in the seminiferous tubule were determined and were compared if there were relationships between them. Comparison between RSBN1 and methylated H4K20 was analyzed at the stages VI-VIII except for elongated spermatids, because RSBN1 was extensively highly expressed in both nucleus and cytoplasm of elongated spermatids and the strong signal impeded comparative analysis between RSBN1 and methylated H4K20 in round spermatids and spermatocytes. As a result, H4K20me3 was accumulated at the heterochromatic region of the nucleus of round spermatids and co-localized with RSBN1 (Fig 4). H4K20me2 were not detected or weak in RSBN1-positive round spermatids at the stages VI-VII and VII-VIII, but H4K20me1 was accumulated in RSBN1-positive round spermatids except for the center of the nucleus of round spermatids (Fig 4). These results suggested that RSBN1 could demethylate H4K20me3 and H4K20me2 into H4K20me1 in round spermatids.

## Discussion

RSBN1 was first reported as a testis-specific gene and its expression was restricted in round spermatids during spermatogenesis [39]. In contrast to the previous report, RSBN1 was not specifically expressed in testis and was expressed not only in round spermatids but also in elongated spermatids at extensively high level in our study. The reason why results of RSBN1 expression between these two reports were different could be hard to be solved but might be the activity and the specificity of the antibody. Bands of immunoblot results were very weak in the previous report, whereas bands of our immunoblot results were quite obvious. On the other hand, immunostaining experiments showed different localization patterns of RSBN1 between the previous study and the current study. These differences might cause a fixation method between two studies. Testes sections were fixed by 4% PFA in PBS in our experiments but were fixed by 100% ethanol in the previous analysis. This experimental difference could lead to distinct results because there are distinct effects against antigens between PFA fixation and ethanol fixation.

RSBN1 was expressed not only in testis but also in brain and ovary. RSBN1 could play a role in meiotic progression and/or post-meiotic progression so that it might have function in oogenesis also. On the other hand, RSBN1 could be involved in the brain development and function and neuronal cell regulation, since H4K20 demethylase PHF8 regulates neuronal cell survival and brain development and another H4K20 demethylase LSD1n regulates memory formation [31,32].

When the demethylation function of RSBN1 was examined, RSBN1 could demethylate not only H4K20me2 but also H4K20me3 in our study. DPY-21, which is the *C*. *elegans* ortholog of RSBN1, catalyzes demethylation of H4K20me2 but not H4K20me3 [22]. In the same report, the molecular function of RSBN1, but partial amino acids of RSBN1 (RSBN1^350-795^), was also analyzed and RSBN1 was shown to demethylate H4K20me2 but not H4K20me3 by *in vitro* experiments. In our study, full amino acids of RSBN1 (RSBN1^1-795^) but not a part of RSBN1 (RSBN1^350-795^) was used to verify the demethylation activity of RSBN1. The N-terminus region of RSBN1 (RSBN1^1-350^) might affect substrate recognition activity of RSBN1 or might be a region to interact with co-factors to demethylate H4K20me3. As shown in our immunostaining results, furthermore, RSBN1 could be responsible for the demethylation of H4K20me3, because H4K20me3 signal was weakened at the stage VII in which expression of RSBN1 in round spermatids was increased. Moreover, H4K20me3 signal was restricted in the center of nucleus of round spermatids and RSBN1 was localized whole nucleus except for the heterochromatic region of round spermatids at the stage VI-VII (Fig 4A) and in the perinuclear region of round spermatids at the stage VIII (Fig 4B). Thus, RSBN1 could be a demethylase for both H4K20me2 and H4K20me3.

Our results suggested that RSBN1 could convert H4K20me3 and H4K20me2 to H4K20me1 but could not demethylate H4K20me1 so that distinct distribution of methylated H4K20 in post-meiotic germ cells could be constructed. Dynamic distributions of methylated H4K20 were re-confirmed in the present study, although the roles of methylated H4K20 during spermatogenesis still remain unknown. Because methylated H4K20 is critical for genome integrity including DNA damage repair, the regulation of methylated H4K20 could be essential for spermatogenesis, especially for meiotic progression [13,14]. H4K20me3 marks the pericentric heterochromatin, silences repetitive DNA and transposon [15]. In our study, localization of H4K20me3 was complemented with previous studies, because H4K20me3 was accumulated at the DAPI-positive heterochromatic region of round spermatids. RSBN1 could play a role to restrict H4K20me3 in the heterochromatic region in round spermatids. Therefore, the weakened H4K20me3 in the step 7 spermatids at the stage VII might be a preparative step toward histone removal to remove heterochromatic region but not be a trigger to reactivate silenced genes. Otherwise, H4K20me3 and its regulation could be involved in the regulation of telomere length, since telomerase activity as well as the restoration of the shortened telomere are detected during spermatogenesis [41–43]. These hypotheses could be revealed by a generation of germ cell specific RSBN1 deficient mice.

Whereas H4K20me3 was decreased in HeLa cells expressing RSBN1, H4K20me3 were remained in the heterochromatic region of round spermatids. However, RSBN1 was localized in the nucleus except for the heterochromatic region of round spermatids at the stage VI and localized at the perinuclear region of round spermatids at the stage VIII. Since RSBN1 was not excluded from the heterochromatic region of HeLa cells, heterochromatic region of round spermatids could be different from that of HeLa cells so that H4K20me3 was remained in the heterochromatic region of round spermatids. There might be specific molecules or chromosome structures in round spermatids that could exclude RSBN1 from and maintain H4K20me3 in the heterochromatic region of round spermatids.

In the previous report, H4K20me1 was enriched on hermaphrodite X-chromosome in somatic cells to repress X-linked gene expression and to regulate higher-order chromosome structure of X chromosome during *C*. *elegans* dosage compensation [23–25]. Whereas the enrichment of H4K20me1 on X chromosome of *C*. *elegans* somatic cells is catalyzed by DPY-21, DPY-21 promotes the enrichment of H4K20me1 on autosomes to induce chromosome compaction in germ cells [22]. In our study, H4K20me1 was accumulated in round spermatids as well as compacted nucleus in elongated spermatids, suggesting that the accumulation of H4K20me1 could be promoted by RSBN1 and be required for the preparation of chromosome compaction during post-meiotic germ cell differentiation.

Although some modifiers of H4K20 methylation could be involved in the regulation and the construction of testis-specific distribution of methylated H4K20, little is known about their expression and function during spermatogenesis. Before this study, RSBN1 was the only H4K20 demethylase which expressed in round spermatids. Recently, RAD23A and B (HR23A and B) were reported as a H4K20me1/me2/me3 demethylases [44]. Interestingly, RAD23B deficient mice show impaired embryonic development and a few survived RAD23B deficient mice are male sterility because of the defects in gonocyte development [45]. Therefore, demethylases for H4K20me3 are not only RSBN1 but also RAD23A and B at present. However, RSBN1 but not RAD23A and B could be responsible for demethylation of H4K20me3 in round and elongated spermatids, because H4K20me3 and H4K20me2 but not H4K20me1 were disappeared from round spermatids as shown in our results. Since RAD23A and B can demethylate both H4K20me3 and H4K20me1, they could not generate the methylation pattern shown in round spermatids. Thus, RSBN1 could have an essential role to regulate H4K20 methylation during spermatogenesis. A mouse model deficient for RSBN1 will be useful to reveal the function of RSBN1 as well as the role methylated H4K20 during spermatogenesis.

## Supporting information

S1 Fig Immunoblot evaluation of the specificity of the RSBN1 antibody. A representative immunoblot result to detect GFP-RSBN1-FLAG protein expressed in culture cells using anti-RSBN1, anti-GFP, and anti-FLAG antibodies.
(TIF)

S2 Fig Immunostaining evaluation of the specificity of the RSBN1 antibody. Representative immunofluorescence images of RSBN1 (red) and GFP (green) in GFP-RSBN1-FLAG expressed culture cells are shown.
(TIF)

S3 Fig Localization of RSBN1 at the stages VIII-IX and XI seminiferous tubules. Low magnification images (a-h) and High magnification images (a’-h’) of the localization of nucleus (DAPI, blue: a, a’, e, and e’), acrosome (PNA, white: b, b’, f, and f’), RSBN1 (magenta, c, c’, g, and g’), and merged images (d, d’, h, and h’) in the seminiferous tubule at stage VIII-IX (a-d and a’-d’) and the stage XI (e-h, and e’-h’) are shown. Scale bars = 50μm (a-h) and 10μm (a’-h’).
(TIF)

S4 Fig (TIF)

S5 Fig (TIF)

S6 Fig (TIF)

S7 Fig (TIF)

S8 Fig (TIF)

S9 Fig (TIF)

S10 Fig (TIF)

S11 Fig (TIFF)

S12 Fig (TIFF)
