# Supplementary figures and images for: Comparative distributions of RSBN1 and methylated histone H4 Lysine 20 in the mouse spermatogenesis
Source: PLoS One. 2021 Jun 29;16(6):e0253897. doi: 10.1371/journal.pone.0253897 (PMC8241091; doi:10.1371/journal.pone.0253897)

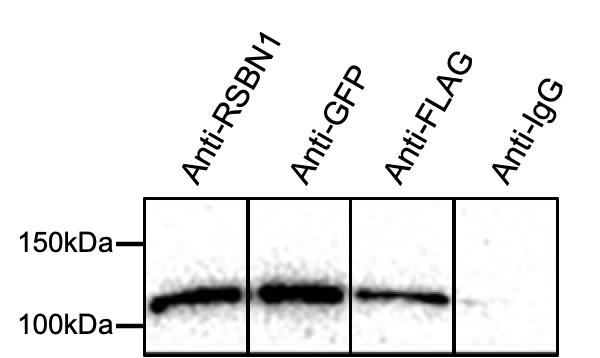

Supplement: S1 Fig — A representative immunoblot result to detect GFP-RSBN1-FLAG protein expressed in culture cells using anti-RSBN1, anti-GFP, and anti-FLAG antibodies. (TIF) [file pone.0253897.s001.tif]

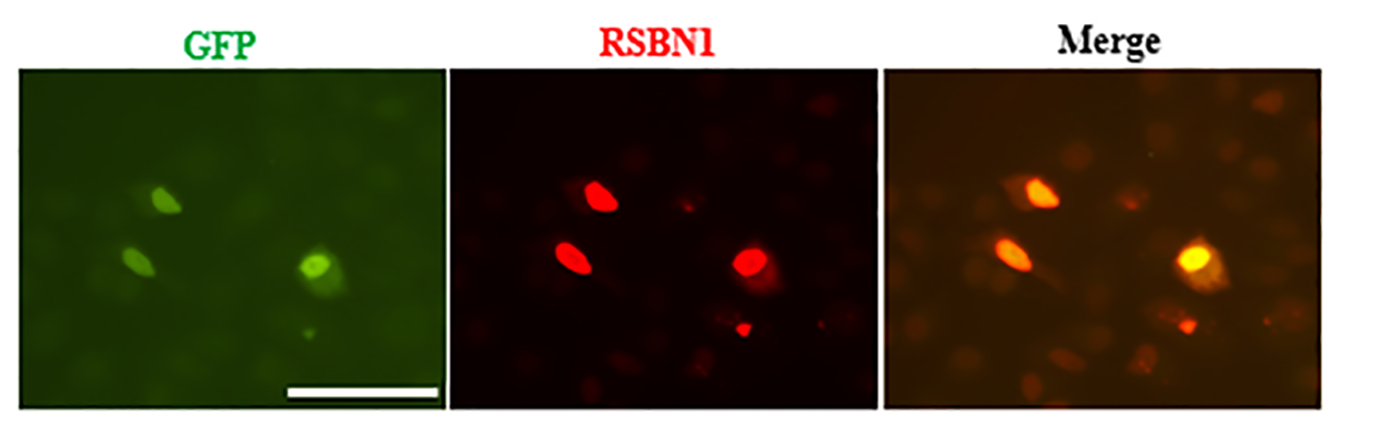

Supplement: S2 Fig — Representative immunofluorescence images of RSBN1 (red) and GFP (green) in GFP-RSBN1-FLAG expressed culture cells are shown. (TIF) [file pone.0253897.s002.tif]

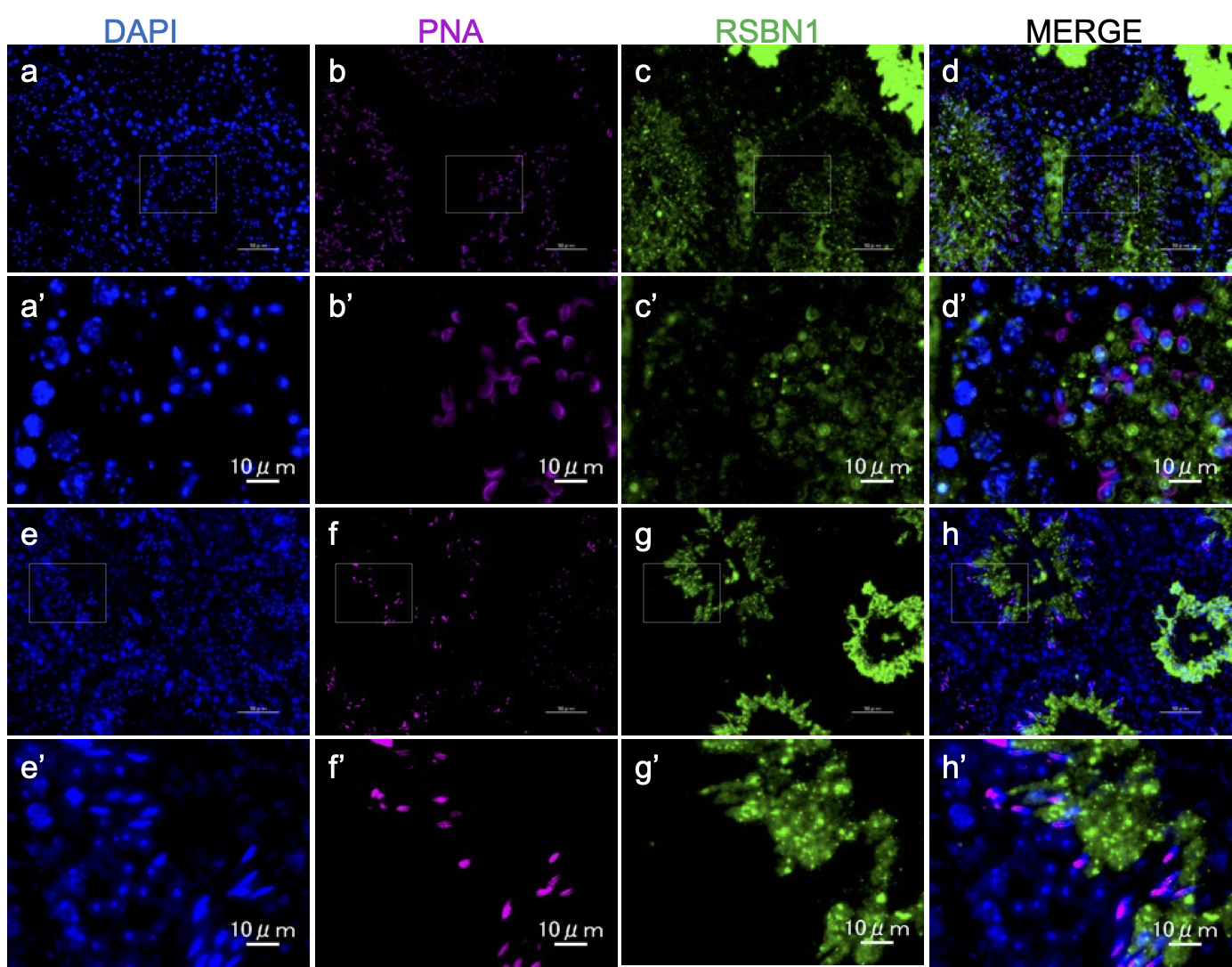

Supplement: S3 Fig — Low magnification images (a-h) and High magnification images (a’-h’) of the localization of nucleus (DAPI, blue: a, a’, e, and e’), acrosome (PNA, white: b, b’, f, and f’), RSBN1 (magenta, c, c’, g, and g’), and merged images (d, d’, h, and h’) in the seminiferous tubule at stage VIII-IX (a-d and a’-d’) and the stage XI (e-h, and e’-h’) are shown. Scale bars = 50μm (a-h) and 10μm (a’-h’). (TIF) [file pone.0253897.s003.tif]

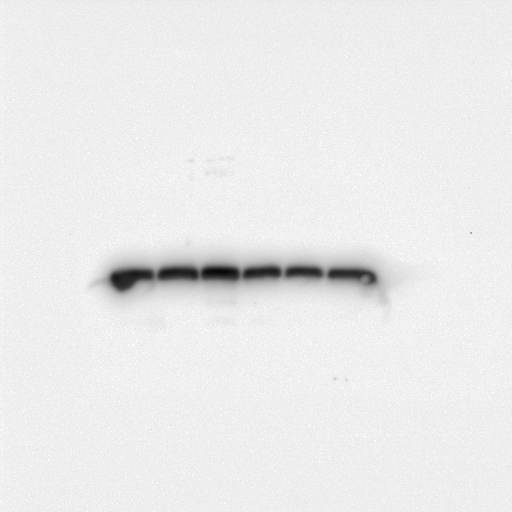

Supplement: S4 Fig — (TIF) [file pone.0253897.s004.tif]

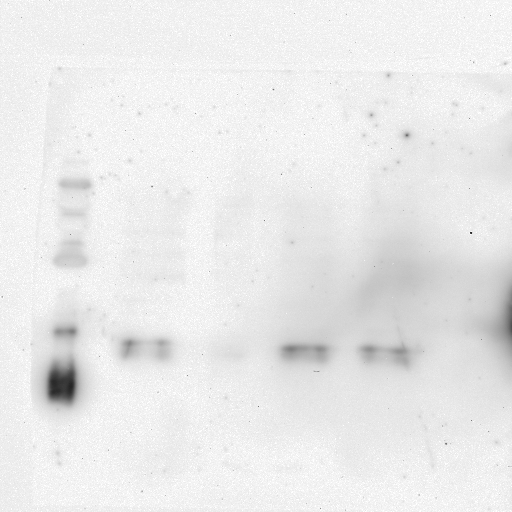

Supplement: S5 Fig — (TIF) [file pone.0253897.s005.tif]

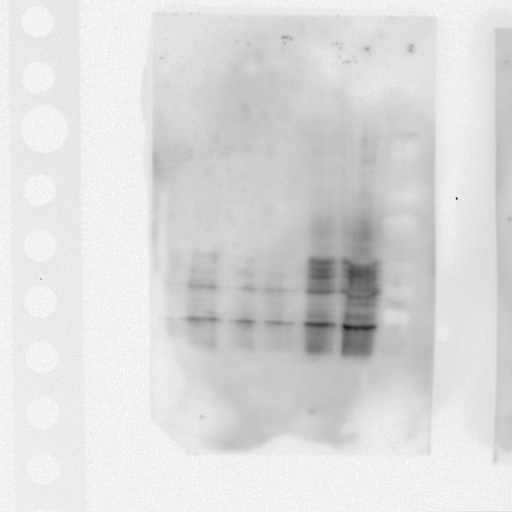

Supplement: S6 Fig — (TIF) [file pone.0253897.s006.tif]

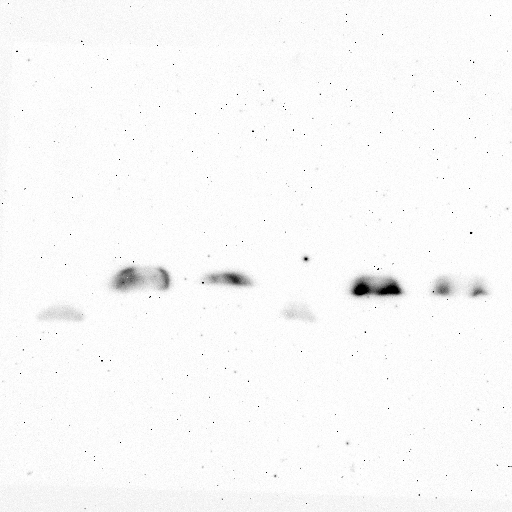

Supplement: S7 Fig — (TIF) [file pone.0253897.s007.tif]

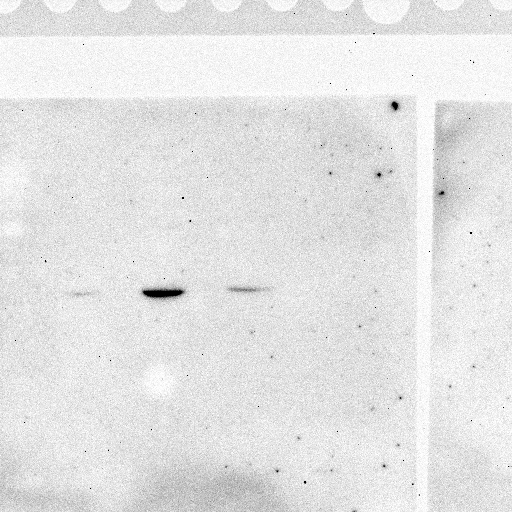

Supplement: S8 Fig — (TIF) [file pone.0253897.s008.tif]

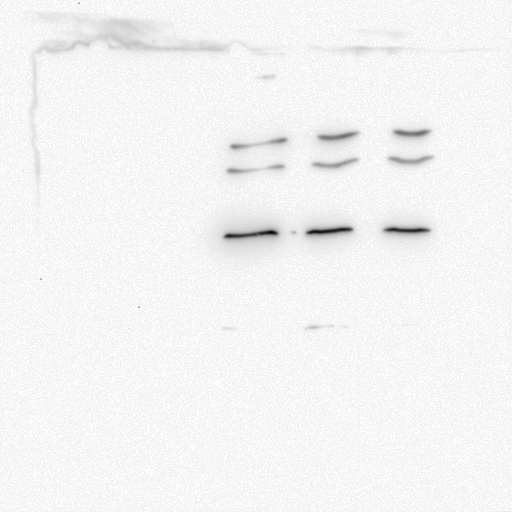

Supplement: S9 Fig — (TIF) [file pone.0253897.s009.tif]

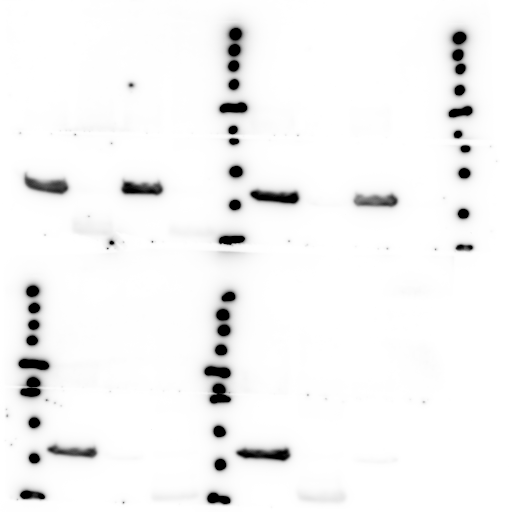

Supplement: S10 Fig — (TIF) [file pone.0253897.s010.tif]

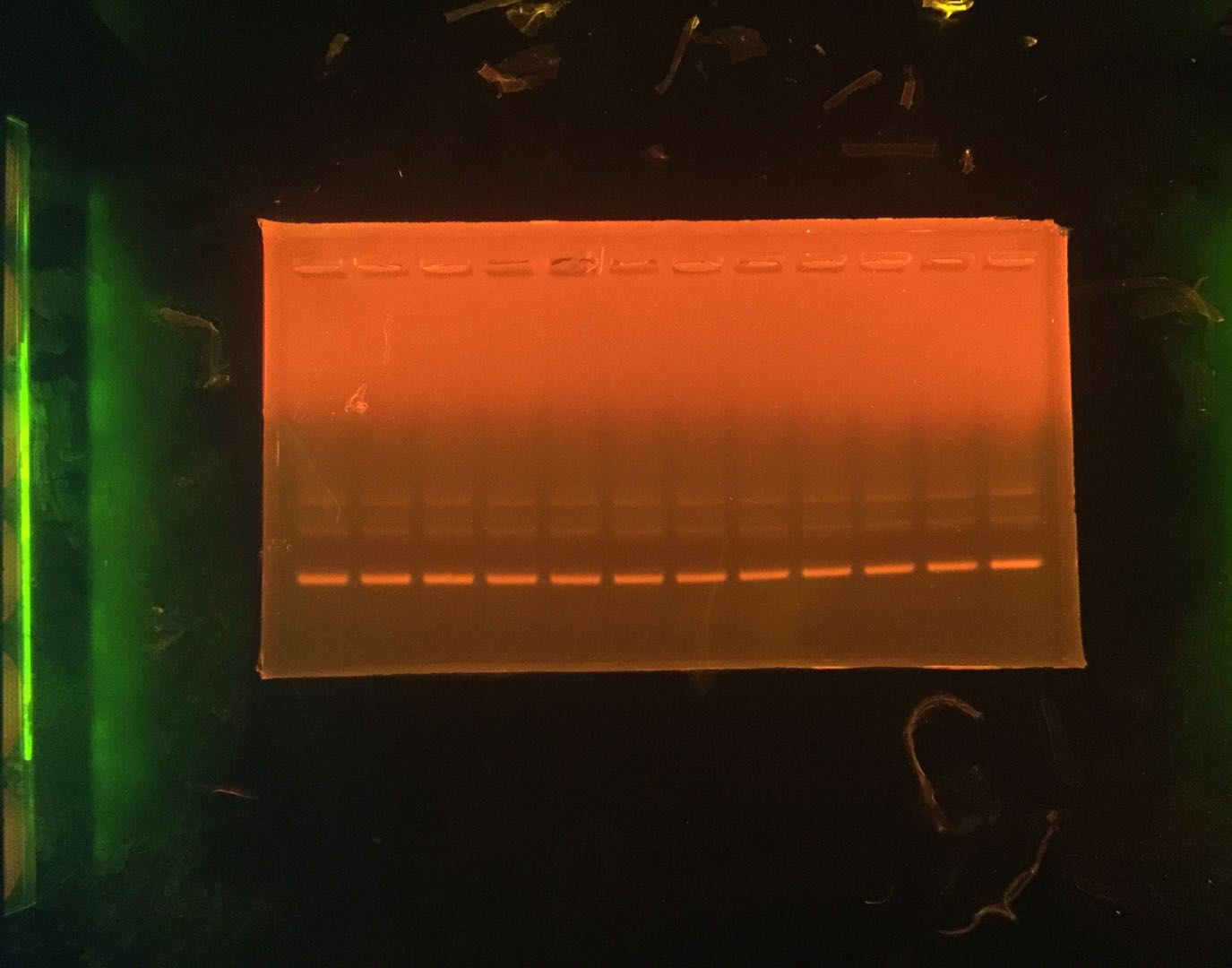

Supplement: S11 Fig — (TIFF) [file pone.0253897.s011.tiff]

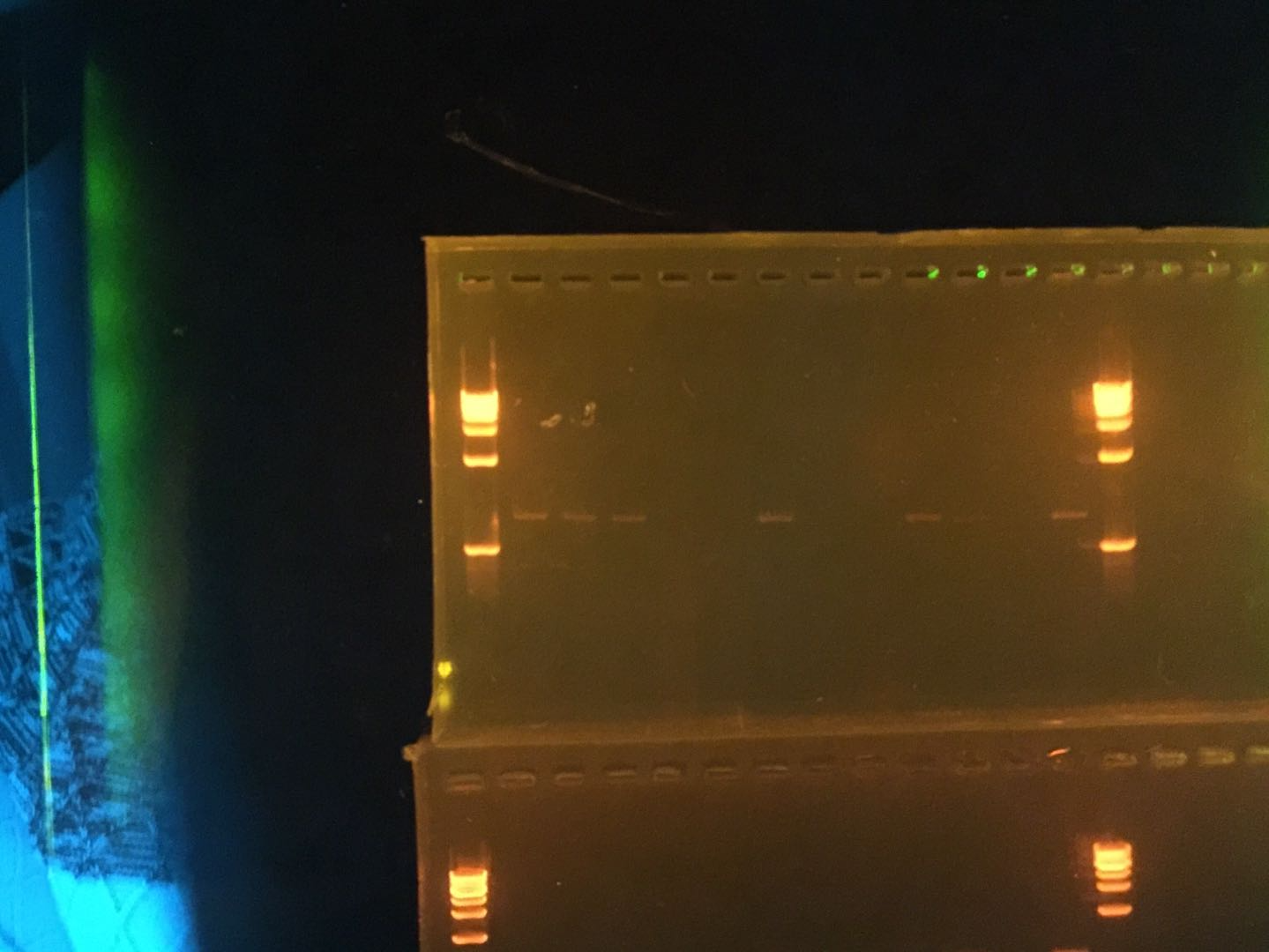

Supplement: S12 Fig — (TIFF) [file pone.0253897.s012.tiff]
